# Modulations in the offspring gut microbiome are refractory to postnatal synbiotic supplementation among juvenile primates

**DOI:** 10.1186/s12866-018-1169-9

**Published:** 2018-04-05

**Authors:** Ryan M. Pace, Amanda L. Prince, Jun Ma, Benjamin D. W. Belfort, Alexia S. Harvey, Min Hu, Karalee Baquero, Peter Blundell, Diana Takahashi, Tyler Dean, Paul Kievit, Elinor L. Sullivan, Jacob E. Friedman, Kevin Grove, Kjersti M. Aagaard

**Affiliations:** 1 Department of Obstetrics and Gynecology, Division of Maternal-Fetal Medicine, Baylor College of Medicine, Houston, TX 77030 USA; 2 Department of Molecular and Cell Biology, Baylor College of Medicine, Houston, TX 77030 USA; 3 Department of Molecular and Human Genetics, Baylor College of Medicine, Houston, TX 77030 USA; 4 Oregon National Primate Research Center, Oregon Health & Science University, Beaverton, OR 97239 USA; 5 Biology Department, University of Portland, Portland, OR 97203 USA; 6 Department of Pediatrics, University of Colorado Anschutz Medical Campus, Aurora, CO USA

**Keywords:** Probiotic, synbiotic, Non-human primate, Microbiome, Dysbiosis

## Abstract

**Background:**

We and others have previously shown that alterations in the mammalian gut microbiome are associated with diet, notably early life exposure to a maternal high fat diet (HFD). Here, we aimed to further these studies by examining alterations in the gut microbiome of juvenile Japanese macaques (*Macaca fuscata*) that were exposed to a maternal HFD, weaned onto a control diet, and later supplemented with a synbiotic comprised of psyllium seed and *Enterococcus* and *Lactobacillus* species.

**Results:**

Eighteen month old offspring (*n* = 7) of 36% HFD fed dams were fed a control (14% fat) diet post weaning, then were synbiotic supplemented for 75 days and longitudinal stool and serum samples were obtained. All stool samples were subjected to 16S rRNA metagenomic sequencing, and microbiome profiles and serum lipids and triglycerides were compared to untreated, healthy age matched and diet matched controls (*n* = 7). Overall, 16S-based metagenomic analysis revealed that supplementation exerted minimal alterations to the gut microbiome including transient increased abundance of *Lactobacillus* species and decreased abundance of few bacterial genera, including *Faecalibacterium* and *Anaerovibrio*. However, serum lipid analysis revealed significant decreases in triglycerides, cholesterol, and LDL (*p* < 0.05). Nevertheless, supplemented juveniles challenged 4 months later were not protected from HFD-induced gut dysbiosis.

**Conclusions:**

Synbiotic supplementation is temporally associated with alterations in the gut microbiome and host lipid profiles of juvenile Japanese macaques that were previously exposed to a maternal HFD. Despite these presumptive temporal benefits, a protective effect against later HFD-challenge gut dysbiosis was not observed.

**Electronic supplementary material:**

The online version of this article (10.1186/s12866-018-1169-9) contains supplementary material, which is available to authorized users.

## Background

Initial studies of the human microbiome characterized the diversity of the healthy gut microbiome, and subsequent analyses have revealed that the gut microbiome is significantly altered in association with obesity, diet, and metabolic disease [1–9]. Similar trends have been observed in animal model systems [10–17], including Japanese macaques (*Macaca fuscata*) [18]. We have previously demonstrated that the gut microbiome of the Japanese macaque is structured similarly to the human gut microbiome and is modulated primarily by diet, which provides an animal model for examining the human microbiome [18]. However, while diet is associated with significant alterations in the gut microbiome [4–9, 12–17, 19], duration and modulation of the offspring gut dysbiosis associated with maternal high-fat diet (HFD) exposure is relatively unexplored. Similarly, while no human studies to date have shown causation of metabolic disease by the gut microbiome, there have been numerous studies associating dysbiosis of the gut microbiome with metabolic disease [2, 3, 11, 12, 20–23]. We have previously shown that exposure to a maternal HFD during gestation and lactation is associated with impaired islet vascularization, increases in hepatic lipids, and dysbiosis of the offspring gut microbiome at 1 year of age [18, 24, 25]. Altogether, this model provides a unique opportunity to closely examine interactions between dietary exposures, metabolic disease, and the microbiome.

Probiotics are any viable microorganisms (e.g. *Lactobacilli and Bifidobacteria*) that when consumed or applied confer beneficial effects to the host [26, 27]. Prebiotics are non-host digestible food substances that act as an energy source for beneficial microorganisms in the gut and encourage microbial colonization, growth, and activity [27]. Synbiotics are a combination of the two. These classes of food products are largely accepted as a non-harmful means to promote metabolic health [27]. Previous studies have indicated that prebiotic, probiotic, and synbiotic supplemental use are associated with alterations in physiologic and metabolic measures of host and/or microbe, bowel function, mucosal contact time, and microbial abundances [28–36]. More specifically, a study with rats demonstrated decreases in weight gain, blood glucose and insulin, and liver triglycerides associated with synbiotic supplementation [33]. Inherent to this notion of benefit is the assumption that the gut is colonized by these probiotic strains and species, although evidence demonstrating either transient or persistent colonization is lacking.

We hypothesized that synbiotic supplementation may modulate the gut microbiome of offspring exposed to a maternal HFD in our non-human primate model (NHP) [18, 37–39]. To test this hypothesis, we utilized a synbiotic composed of probiotic species of *Enterococcus* and *Lactobacillus* and a prebiotic of psyllium seed. Psyllium seed (*Plantago ovata*), is composed mainly of a highly branched arabinoxylan [40], and is a prevalent prebiotic [35, 41, 42]. Psyllium seed is not absorbed into the bloodstream; instead, it passes through the gastrointestinal tract and binds with water to increase stool bulk. Thus, psyllium seed is used to treat diarrhea and diarrhea-like symptoms by absorbing and retaining water in order to make stool more formed and less fluid [43, 44]. In concordance, psyllium seed has been used clinically with diarrhea, irritable bowel syndrome, and inflammatory bowel diseases (e.g. ulcerative colitis and Crohn’s disease; reviewed in [45, 46]). Here, we aimed to determine in our NHP model if synbiotic use leads to transient alterations in the gut microbiome and serum lipid profiles [37–39]. Additionally, and in accordance with our NHP maternal model of obesity, we aimed to examine if synbiotic treatment modulated dysbiosis of the gut associated with a short term HFD challenge at 2 years of age and following 18 months of control diet feeding.

## Methods

### Experimental design

**Fig. 1:**
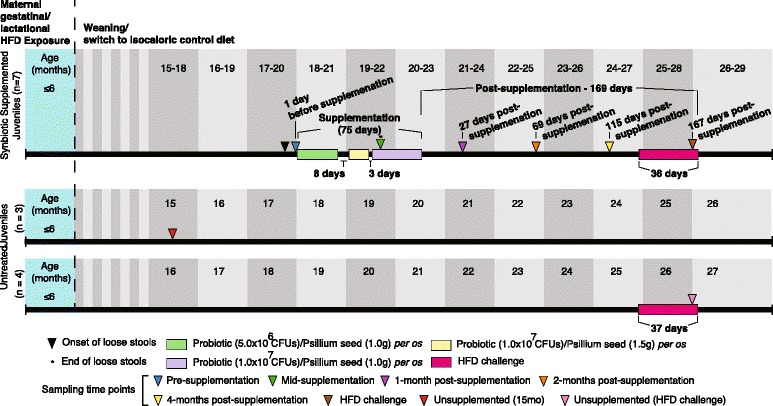
Experimental timeline summary. Timeline of sample acquisition, synbiotic supplementation, and high-fat diet challenge. Around 18 months of age a small group of juveniles (*n* = 7) were noted to have loose stools of unknown origin and supplementation with a synbiotic was initiated within a week of symptom onset. Supplementation lasted for 75 days and consisted of psyllium seed and the following probiotic bacteria: Enterococcus faecium, Lactobacillus acidophilus, Lactobacillus plantarum, and Lactobacillus casei. On day 50 (denoted by the asterisk) of the 75-day synbiotic supplementation period the majority juvenile’s stools (*n* = 5) were observed to return to normal. In addition to synbiotic supplementation, juveniles were HFD challenged at 23 to 24 months of age. Days and months post-supplementation is counted from the time that supplementation ended.

The use of *Macaca fuscata* by our group of investigators has been previously described [18, 37–39, 47–50]. In brief, animals were socially housed within indoor and outdoor enclosures at the Oregon National Primate Research Center (ONPRC). All methods were carried out in accordance with IACUC guidelines and regulations, and all experimental protocols were approved by the IACUC at both ONPRC and Baylor College of Medicine. Dams were maintained on a high-fat diet (HFD) consisting of 36% fat (from porcine and poultry fat, corn and fish oil; TAD Primate Diet – 5LOP, Test Diet, St. Louis, MO) that is supplemented with calorically dense treats (consisting of Glaxo powder/TAD pellets, peanut butter, honey, banana, and cornstarch). Animals bred naturally, and in the current study cohort (Fig. 1) offspring were maintained on the same HFD as the dams (via lactation) until weaning at 7 months of age. Post weaning, the offspring were switched to an isocaloric control diet consisting of 14% fat (from soybean oil, Fiber-Balanced Monkey Diet 5052, Lab Diet, St. Louis, MO).

In this opportunistic study, one socially housed troop of juvenile offspring (17–20 months of age) was observed to have intermittent non-infectious loose stools (culture negative, absence of clinical disease including absence of hair and weight loss, social irritability, or appetite loss) but did not experience any weight loss during the duration of the study (Additional file 1: Figure S1). Within 7 days the animals were supplemented with synbiotic therapy. The synbiotic formulation was administered per os over the course of 75 days hidden in daily treats (consisting of banana, yogurt, pumpkin puree, oats, cinnamon, water, honey, gelatin, probiotic, and prebiotic; Fig. 1). The probiotic component of the synbiotic consisted of 5.0 × 10^6^–1.0 × 10^7^ CFUs of *Enterococcus faecium* (*SF-273*), *Lactobacillus acidophilus* (*LC-322*), *Lactobacillus plantarum* (*LA-5*), and *Lactobacillus casei* (*CH6072*) (Probiocin oral pet gel, Probios). The prebiotic component consisted of 1.0–1.5 g of psyllium seed powder (EquiAid Natural Psyllium Fiber, Phoenix, AZ; Fig. 1). Around 27 months of age, and post-supplementation, juveniles were challenged with a HFD for 36 days. Two juvenile cohorts that were untreated, healthy, age and dietary exposure matched were used for comparative analyses, including one sample set corresponding to the pre-supplementation time point (*n* = 3, two males and one female, 15 months old) and another sample set during a HFD challenge time point (*n* = 4, all females, 25–27 months old). Animals utilized in this study were not treated with antibiotics at any point during the course of the study.

### Blood (serum lipid) analysis

For triglycerides, cholesterol, LDL, and HDL levels, blood samples were taken prior to the start of the synbiotic supplementation and at one-month post-supplementation and submitted for serum lipid profile analysis. Whole-blood samples were collected in red top Vacutainer collection tubes. Serum was separated from whole blood by centrifugation at 2400 rpm for 6 min (Eppendorf Centrifuge5810, Westbury, New York). Biochemistry and lipid profiles were performed on an ABXPentra400 (Horiba, Irvine, CA) in accordance with manufacturer’s guidelines. Quality control procedures were performed each morning prior to analysis with commercial QC materials and samples were run only when the control runs passed inspection. An aliquot of 300 μl was analyzed for cholesterol, triglyceride, high- and low-density lipoproteins. Statistical significance was determined using the Wilcoxon matched-pairs signed rank test (non-parametric).

### Sequencing of the microbiome

When animals were individually caged for procedures, samples were collected both as stool and anal collections via cotton swabbing at several time points, including prior to the start of the synbiotic supplementation (17–20 months old, anus), midway through supplementation (stool), and post-supplementation [corresponding to one (stool), two (anus, stool), and 4 months (stool)] and during a HFD challenge that occurred 5 months after treatment (stool, 26–28 months old; Fig. 1, Additional file 2: Table S1). Additionally, samples were collected from the healthy, age and dietary matched, offspring at separate time points: 15 months of age (anal and stool swabs) and during a HFD challenge (anal swabs, 25–27 months old; Fig. 1, Additional file 2: Table S1). Microbial DNA was isolated via the PowerSoil DNA Isolation Kit (MOBIO, Carlsbad, CA). After DNA extraction, microbial 16SrDNA was amplified using V3 V5 primers with barcodes attached for sample identification before Roche454 sequencing [18, 51]. The V3 V5 primer sequences used include, with barcodes in lower case letters: 5’-cctatcccctgtgtgccttggcagtctcaGCCTACGGGAGGCAGCAG-3′ (B-354F forward primer) and 5’-ccatctcatccctgcgtgtctccgactcagNNNNNCCGTCAATTCMTTTRAGT-3′ (A-926R reverse primer).

### 16S data analysis

Sample reads were analyzed using quantitative insights into microbial ecology (QIIME, version 1.9.1) [52]. Briefly, reads lacking a barcode or primer sequence, reads with length less than 200 nucleotides, and reads with a minimum average quality score less than 25 were removed. De-noised sequences were clustered into operational taxonomic units (OTUs) at the 97% similarity level through closed-reference OTU picking using the Greengenes13.5 database [53], resulting in 73% of sequences (316,470/432,779) remaining for subsequent analysis (Additional file 2: Table S1). Microbial diversity was evaluated within samples (alpha diversity) or between samples (beta diversity) on rarefied OTU tables. Rarefaction resulted in 2969 and 3125 sequences from each sample used in the analyses of the intervals containing the synbiotic supplementation and HFD challenge, respectively. Alpha diversity was evaluated using Shannon Diversity Index and chao1. Beta diversity was evaluated using phylogenetic distance (weighted and unweighted UniFrac) [54]. The resulting distance matrices served as inputs for principal coordinates analysis (PCoA) and significance of sample clustering by PERMANOVA with 999 permutations. Phylogenetic investigation of communities by reconstruction of unobserved states (PICRUSt) [55] was used to infer relative abundance of bacterial encoded metabolic pathways, as defined by the Kyoto Encyclopedia of Genes and Genomes (KEGG;http://www.genome.jp/kegg/). Significant differences in bacterial taxa and metabolic pathways between groups was identified via STAMP (White’s non-parametric t-test for two groups comparisons) [56] and feature selection through linear discriminant analysis (LDA) effect size (LEfSe) [57]. For LEfSe, the alpha value for the factorial Kruskal–Wallis test was 0.05 and the threshold on the logarithmic linear discriminant analysis (LDA) score for discriminative features was 2.0. Weight and lipid data were analyzed using Prism 6 (GraphPad Software Inc La Jolla, CA). The 16S sequence data generated from this analysis have been deposited in the Sequence Read Archive (SRA) under accession bioproject ID PRJNA317339.

### Probiotic species-specific PCR

Primers were selected from previously reported studies [58, 59] or designed using Primer-BLAST from the National Center for Biotechnology Information (NCBI) [60]. Primer sequences and annealing conditions are included in Additional file 3: Table S2. Resultant amplicons were run on a 2% agarose gel and visualized using a GelDoc-it [2] Imager (UVP, LLC Upland, CA). For quantitative real-time PCR (qPCR), the FastSYBRGreen kit (Applied Biosystems ABI, Waltham, MA) was utilized, and the reaction was run on a StepOnePlus PCR System (ABI). The following strains of the probiotic species were used as positive controls for PCR - *L. casei* SD5868, *L. acidophilus* ATCC521, and *L. plantarum* ATCC14917.

## Results

### Alterations of the juvenile gut microbiome are associated with synbiotic supplementation

**Fig. 2:**
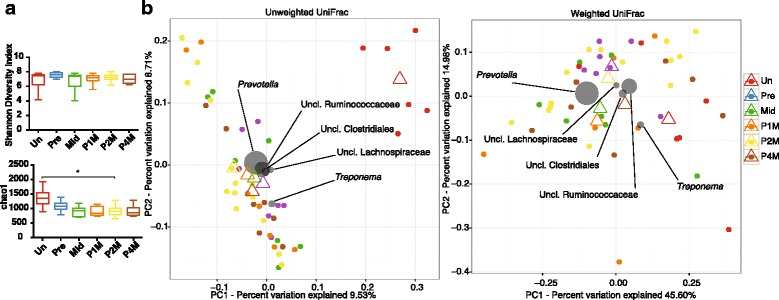
Minimal significant alterations in the gut microbiome are associated with synbiotic supplementation in juvenile Japanese macaques. To reduce sampling heterogeneity, rarefaction was performed and resulted in 2969 sequences from each sample in this analysis. **a** Alpha diversity using the Shannon Diversity Index is similar between untreated juveniles at 12 months of age and synbiotic supplemented juveniles during treatment, but significantly lower by chao1. Statistical significance was tested using Dunn’s multiple comparisons test (*p ≤ 0.05). **b** PCoA displaying beta diversity of untreated juveniles at 12 months of age in comparison to treated juveniles at pre-supplementation, mid-supplementation, and post-supplementation (1, 2, and 4 months). Gray circles represent relative contributions in sample clustering for the top five genera. Open triangles represent centroids and ellipses indicate 95% confidence intervals. The juvenile cohort that received synbiotic supplementation was found to cluster separately from the cohort that did not receive synbiotic treatment (unweighted UniFrac PERMANOVA *p* = 0.001; weighted UniFrac PERMANOVA *p* = 0.005). Abbreviations: untreated (no supplementation) 12-month-old juveniles (Un) and synbiotic supplemented juveniles at pre-supplementation (Pre), mid-supplementation (Mid), and post-supplementation (one, two & 4 months: P1M, P2M & P4M)

**Fig. 3:**
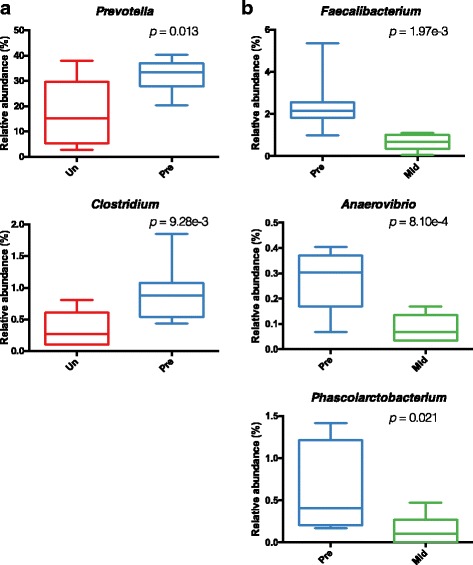
Synbiotic supplementation is associated with trends in genus level taxonomic changes. LEfSe analysis was utilized at the genus level to identify alterations in taxonomical enrichment between (**a**) animals that were untreated and prior to synbiotic supplementation, and between (**b**) animals prior to synbiotic supplementation and during mid-supplementation. Abbreviations as in Fig. 1. Statistical significance was tested using Dunn’s multiple comparisons test (**p ≤ 0.01)

Examining taxonomic differences between untreated juveniles and synbiotic supplemented juvenile offspring (Fig. 1), we identified shifts in bacterial taxa (Fig. 2). Examination of alpha diversity measures (i.e.*,* within sample diversity) among offspring that received synbiotic supplementation at pre, mid, and post-supplementation (1, 2 & 4 months after supplementation ended) indicated unchanged Shannon but significantly lower chao1 metrics (Fig. 2a). By beta diversity metrics, untreated juveniles clustered separately from the synbiotic supplemented juveniles at all stages of synbiotic treatment but not pretreatment (unweighted UniFrac PERMANOVA *p* = 0.001; weighted UniFrac PERMANOVA *p* = 0.005; Fig. 2b, Additional file 4: Table S3). These could largely be attributed to a few taxa, including *Prevotella* (0.17% in untreated animals and 0.32% in animals prior supplementation, *p* = 0.013) and *Clostridium* (untreated 0.003% vs pre-supplementation 0.009%, *p* = 9.28e^− 3^; Fig. 3a, Additional file 5: Figures S2 & S3). This data is in general agreement with previous reports on the association of *Prevotella* with high fiber diets [4, 6, 19], which the psyllium would provide. This is further consistent with our observed significant shifts in the relative abundance at the genus level as identified via LEfSe analyses, including decreases in *Faecalibacterium* (from 2.46 to 0.63%, *p* = 1.97e^− 3^), *Anaerovibrio* (from 0.27% to 0.09%, *p* = 8.10e^− 4^), and *Phascolarctobacterium* (from 0.65% to 0.15%, *p* = 0.021; Fig. 3b, Additional file 5: Figures S2 & S3). A decrease in *Anaerovibrio* is in accordance with a prior study where *A. lipolytic* was decreased in association with a high-fiber diet [61]. Of note, we did not detect significant changes in the bacterial taxa related to the probiotic species during treatment, including manual inspection of the *Enterococcus* and *Lactobacillus* OTU reference sequences (with > 10 read counts) from the untreated, pre-, mid-, and post-supplementation (Additional file 6: Figure S3B and Additional file 7: Table S4). While manual inspection of these samples revealed three putative probiotic OTUs with blast hits against the probiotic species, these OTUs were either not detected in the mid-supplementation samples or were not significantly increased compared to pre-supplementation (Additional file 7: Table S4). Furthermore, we were only able to detect one of the four probiotic species (*L. acidophilus*) in the synbiotic supplemented animals by PCR (Additional file 8: Figure S4) but did detect overall changes in the *Lactobacillus* genus via quantitative real-time PCR (qPCR, Additional file 9: Figure S5).

### Synbiotic supplementation is temporally associated with improvements in serum lipids and associated bacterial metabolic pathways

**Fig. 4:**
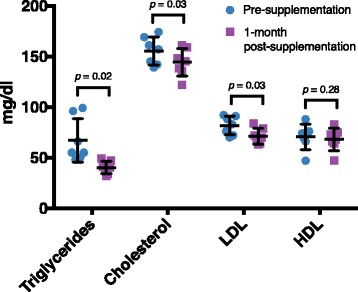
Host lipid profiles are altered in association with synbiotic supplementation. Juvenile offspring triglycerides, cholesterol, and LDL (but not HDL) profiles were significantly decreased at one-month post-supplementation in comparison to pre-supplementation

Despite the relatively minimal change in bacterial taxa associated with synbiotic supplement, we did find significant alterations in host lipid profiles (Fig. 4). Analysis of serum taken at pre-supplement and one-month post-supplement revealed significant decreases in triglycerides (67.0 to 40.1 mg/dl, *p* = 0.02), cholesterol (155.3 to 144.1 mg/dl, *p* = 0.03), and LDL levels (81.7 to 71.3 mg/dl, *p* = 0.03). In contrast, although lowered, we did not find a significant difference in the level of HDL (70.4 to 68.0 mg/dl, *p* = 0.28). However, despite the statistical significance of these variations, the physiologic and clinical significance is non-evident as the mean variation (in mg/dL) was within ~ 10% of initial values. In contrast, serum triglyceride levels demonstrated a higher degree of variance pre and post synbiotic supplementation (59.8% of the initial value, Fig. 4). Over the course of this study we did not detect any significant changes in the weight of the animals that received synbiotic supplementation at any time point prior or during supplementation, nor in comparison to untreated, age and dietary-matched animals (Additional file 1: Figure S1). On day 50 of the 75-day synbiotic supplementation the majority of the juveniles (5 out of 7; 71.4%) were observed to have normal, firm stool.

**Fig. 5:**
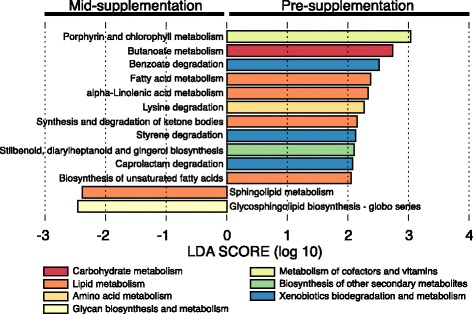
Shifts in inferred metabolic pathways are associated with synbiotic supplementation. LEfSe plot of inferred KEGG bacterial metabolic pathways with differential abundance. Bars to the left indicate inferred bacterial metabolic pathways that are enriched in animals during synbiotic supplementation, while bars to the right indicate inferred bacterial metabolic pathways that are enriched in animals during pre-supplementation. Bar colors indicate higher-level metabolic pathways

With these temporal lipid modifications in mind, we further examined inferred bacterial metabolic pathways for alterations in association with synbiotic supplementation and did observe significant differences (Fig. 4). Specifically, we observed significant decreases in several lipid metabolism pathways, including fatty acid metabolism (0.22 to 0.20%, *p* = 0.038), alpha-linolenic acid metabolism (0.002 to 0.001%, *p* = 5.18e^− 3^), and synthesis and degradation of ketone bodies (0.04 to 0.03%, *p* = 1.30e^− 3^; Fig. 5, Additional file 10: Figure S6). In contrast, the only metabolic pathways that we observed as enriched during synbiotic supplementation were for sphingolipid metabolism (0.19 to 0.22%, *p* = 0.080) and glycosphingolipid biosynthesis-globo series (0.12 to 0.14%, *p* = 0.070; Fig. 5, Additional file 10: Figure S6).

### Synbiotic treatment does not confer protection from HFD-associated gut dysbiosis following later challenge

**Fig. 6:**
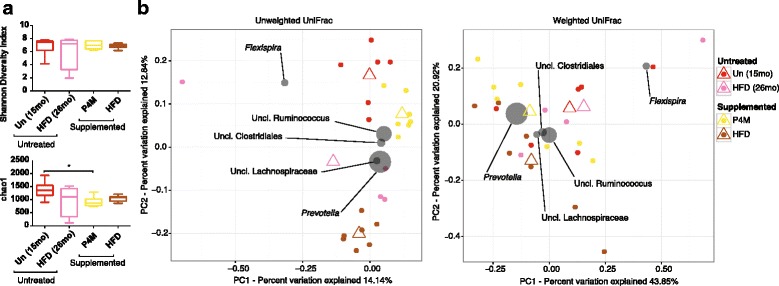
HFD challenge is associated with alterations in the gut microbiome. To reduce sampling heterogeneity, rarefaction was performed utilizing 3125 sequences from each sample. **a** Alpha diversity of synbiotic supplemented juveniles at 4-months post-supplementation and HFD challenge, and untreated juveniles at 15 months and during HFD challenge (26 months). 4-months post-supplementation juveniles were found to have a significant lower alpha diversity compared to untreated juveniles as measured by chao1 but not by Shannon. Statistical significance was tested using Dunn’s multiple comparisons test. Asterisk indicates a *p* ≤ 0.05. **b** PCoA displaying beta diversity (unweighted and weighted UniFrac) of synbiotic supplemented juveniles at 4-months post-supplementation, HFD challenge, and untreated juveniles at 15 months and during HFD challenge (26 months). Gray circles represent relative contributions in sample clustering for the top five genera. Open triangles represent centroids and ellipses indicate 95% confidence intervals. All groups were found to significantly cluster different from one another (unweighted UniFrac PERMANOVA, *p* = 0.001; weighted UniFrac PERMANOVA, *p* = 0.024). The juvenile cohort that received synbiotic supplementation was found to cluster separately from the cohort that did not receive synbiotic supplementation (unweighted UniFrac PERMANOVA, *p* = 0.001; weighted UniFrac PERMANOVA, *p* = 0.022). Abbreviations: Un (15mo): untreated juveniles at 15 months; HFD (26mo): untreated juveniles during HFD challenge at 26 months; P4M: treated juveniles at 4-months post-treatment; HFD: treated juveniles during HFD challenge

**Fig. 7:**
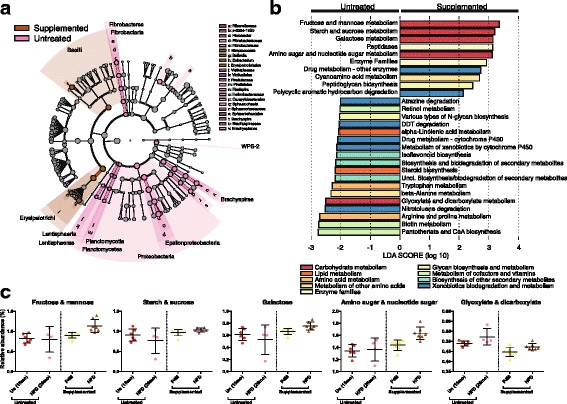
Prior synbiotic supplementation is associated with differences in bacterial taxa and inferred metabolic pathways during HFD challenge. **a** LEfSe generated cladogram of taxonomic differences of abundance. Areas shaded purple indicate bacterial taxa with a higher abundance in prior supplemented animals during HFD challenge, whereas areas shaded green indicate bacterial taxa with a higher abundance in untreated animals during HFD challenge. **b** LEfSe plot of inferred KEGG bacterial metabolic pathways. Bars to the left indicate inferred bacterial metabolic pathways enriched in untreated animals during HFD challenge, whereas bars to the right indicate inferred bacterial metabolic pathways enriched in prior supplemented animals during HFD challenge. Bar colors indicate higher-level metabolic pathways. **c** Plots of the relative abundance of differentially enriched carbohydrate metabolism pathways identified via LEfSe analysis of prior supplemented versus untreated animals during HFD challenge. Abbreviations used as in Fig. 4

Nineteen weeks after the synbiotic supplementation ended, juveniles were challenged for 1 month with the 36% HFD that they had been previously exposed to via their mothers (during both gestation and lactation; Fig. 1). Overall, we observed significant differences in the chao1 but not Shannon alpha diversity measure between groups (Fig. 6a), alongside both unweighted and weighted UniFrac beta diversity (unweighted UniFrac, PERMANOVA, *p* = 0.001; weighted UniFrac, PERMANOVA, *p* = 0.024; Fig. 6b, Additional file 11: Table S5). Deeper taxonomic interrogations via LEfSe revealed several non-significant differences in the relative abundance of bacterial taxa before and during the HFD challenge. *Bacteroidales* (Bacteroidetes), *Fibrobacteres*, and *Spirochaetes* were not significantly reduced (38.9 to 32.3 [*p* = 0.677], 0.53 to 0 [*p* = 4.67e^− 3^], 8.2 to 2.4% [*p* = 0.078], respectively), whereas Tenericutes and Erysipelotrichi were significantly increased (0.3 to 2.5% [*p* = 0.014], 0.28 to 16.2% [*p* = 8.82e^− 4^], Additional file 12: Figure S7). Additionally, and consistent with our prior findings [18], classes of Proteobacteria were non-significantly differentially abundant in association with the postnatal HFD challenge: Epsilonproteobacteria were non-significantly enriched from 0.009 to 0.99% (*p* = 0.150), whereas Deltaproteobacteria were non-significantly reduced from 0.26 to 0.01% (*p* = 0.086) (Additional file 12: Figure S7). Although synbiotic supplementation did not inhibit HFD-associated gut dysbiosis, there were a number of significant differences in comparison to animals that received a HFD challenge but no synbiotic supplementation prior (Fig. 7a, b). In particular, there was a non-significant increased abundance of taxa from Bacilli (*Streptococcus* 2.61 to 0.06%, *p* = 0.102]), Erysipelotrichi (0.11 to 0% *p* = 0.06]); significance was uniquely observed in *Bulleidia* (2.46 to 0.63% *p* = 6.49e^− 3^). The non-significant increase in Erysipelotrichi and decrease in Fibrobacteres appear only among pre/prior synbiotic supplementation state (Additional file 12: Figure S7C). This is particularly potentially intriguing with regard to *Eubacterium* as this genus has been previously associated with obesity and a HFD [62]. Furthermore, during the HFD challenge we found that prior synbiotic supplemented juvenile had an enrichment of pathways for carbohydrate metabolism and enzyme families and reduced enrichment of pathways for xenobiotics biodegradation and metabolism and metabolism of cofactors and vitamins in comparison to untreated juveniles (Fig. 7b, c, Additional file 13: Figure S8).

## Discussion

Overall, we found that there were observable and a few significant alterations in the gut microbial community structure and function (e.g.*,* gut ecology) in temporal association with synbiotic use. These microbiome rearrangements associated with synbiotic supplementation included several shifts in taxonomic relative abundances during synbiotic supplementation which have been associated with host biology. For example, we saw a decrease in *Faecalibacterium*, and *F. prausnitzii* has been associated with anti-inflammatory properties in Crohn’s disease and in animal models of colitis [63, 64]. Similarly, we observed a decrease in the relative abundance of *Phascolarctobacterium*, which has been shown increase in relative abundance in rats that received a HFD compared those on control diet [14]. Lastly, we saw a decrease in *Anaerovibrio*, which is in agreement with a previous study in Surti buffalo associating gut ecology and dietary fiber [61, 65].

Based on the limited but observable and significant taxonomic shifts observed during synbiotic supplementation, it is not surprising that we observed statistically significant concurrent decreases in cholesterol, triglyceride, and LDL levels (Fig. 4). The results of our lipid profiles prompted us to examine the inferred metabolic pathways of the gut microbiome in greater detail, and provide microbiome community function data which correlates well with our serum lipid measurements. Consistent with both our microbiota and host lipid data, we found a decrease in fatty acid metabolism pathway gene content in association with synbiotic exposure (Fig. 5). These data are in concordance with prior studies in rats and humans that have found synbiotic consumption leads to decreased levels of serum cholesterol and triglycerides [66, 67]. However, while we were unable to determine if there was a higher abundance of catabolic versus anabolic pathways, our prior work in this model has shown that lipid metabolism pathways are positively correlated to bacteria, including *Campylobacter* and *Runimococcus* [18]. In the current study, we observed enrichment for sphingolipid metabolism and glycosphingolipid biosynthesis-globo series pathways during synbiotic supplementation. Interestingly, this metabolic pathway data is consistent with previous work of others demonstrating that treatment with a *Bacteroides* derived glycosphingolipid in gnotobiotic mice regulates colonic invariant natural killer T cell (iNKT) homeostasis to protect against oxazolone induced colitis [68]. Taken together, our findings and those of others suggest that synbiotic supplementation may act as an ecological modulator of the gut microbial community, rather than via direct xenobiosis.

Despite these presumptive beneficial findings during and immediately post synbiotic supplementation, we failed to observe persistent significant metabolic protection from gut dysbiosis following later HFD challenge. However, we did observe several changes in bacterial taxa at the genus level between synbiotic treated animals to untreated animals during HFD challenge which are worth commenting on. The lower relative abundance of *Fibrobacter* is likely related to the HFD, as *F. succinogenes* is a well-known degrader of cellulose [69], and cellulose is a principal component of the Japanese macaque diet. The remaining genera that were observed to have a lower relative abundance in synbiotic supplemented animals have been previously associated with various forms of gastrointestinal distress. *Brachyspira* are associated with intestinal spirochetosis and diarrhea [70], and *Sphaerochaeta*, related to *Brachyspira*, have been shown to have highly chimeric genomes, sharing more than 40 % of the total number of its genes with Clostridia [71]. *Flexispira*, reclassified as members of *Helicobacter* [72], are associated with colitis in humans [73]. The bacteria with increased relative abundance in the gut microbiome of synbiotic-supplemented juveniles include *Bulleidia, Eubacterium,* and *Streptococcus. Bulleidia extructa* is an obligate anaerobe that has been described in association with periodontal disease [74]. While *B. extructa* is described as an oral pathogen, it is related to *Holdemania filiformis* and *Erysipelothrix rhusiopathiae,* which are both found in the stool [74]. Therefore, it’s possible that a species of *Bulleidia* also resides in the gut, which is supported by a study examining the microbiome of the appendix [75]. In this study, the relative abundance of *Bulleidia* was increased in subjects with appendicitis [75]. While *Bulleidia* has been associated with disease, it was found in the appendix and rectum of healthy subjects although at a low relative abundance [75]. *Eubacterium* is also associated with host disease and has been found to be increased in coronary heart disease subjects with metabolic syndrome [62]. While *Eubacterium* are one of the most abundant group of human colonic butyrate producers [76], we did not observe a significant difference in the predicted pathway for butyrate metabolism during the HFD challenge. *Streptococcus* species have been implicated in both detrimental and beneficial aspects of non-alcoholic fatty liver disease (NAFLD) [67, 77], and we have previously extensively described that both juveniles and fetuses exposed to a maternal HFD have NAFLD in our Japanese macaque model [25, 38, 47, 78]. While a strain of *S. mutans* has been shown to aggravate NAFLD in mice [77], *S. thermophilus* in a probiotic cocktail mixture that included *Lactobacillus* and *Bifidobacterium* species was shown to ameliorate NAFLD induced by a HFD in rats [67]. While our synbiotic cocktail did not include *Streptococcus* species, we speculate that our findings are consistent with the host having multiple body site-specific niches where one microbe supplementation (or loss) subsequently disrupts the relative abundance of another [1, 51, 79–81].

Accompanying these alterations in several taxa occurring in association with the HFD challenge were modulations in their inferred bacterial metabolic pathways. For example, expression of a glyoxylate shunt in mice resulted in resistance to the associated effects of HFD consumption, such as improved fat mass, leptin, and plasma triglycerides [82]. While the pathway for glyoxylate metabolism was not enriched in the supplemented animals compared to non-supplemented animals, it was increased in response to the HFD challenge in both groups (Fig. 7c). In further agreement, a prior study with rats provided a high prebiotic fiber diet (derived from inulin and oligofructose) post-weaning were shown to have a dampened response to the negative effects associated with HFD consumption (e.g.*,* lower ratio of Firmicutes to Bacteroidetes and decreased cholesterol) [19]. These results were considered to be due to the fiber affecting alterations in the gut microbiota and hepatic gene expression [19]. Taken together, our observations lead us to speculate that the altered carbohydrate metabolic pathways are most likely attributable to the establishment of a microbial community during the synbiotic supplementation that more readily produces short chain fatty acids (SCFAs) [83]. Although we did not measure SCFA production directly, it is well-documented that psyllium alters SCFA production [84] and thereby lipid metabolism [85], leading to an altered response of the microbiome in association with the HFD challenge in the animals that received prior synbiotic supplementation. While we are able to speculate on potential synbiotic and HFD gut taxonomic and functional pathway associations as identified by metagenomic sequencing, future in-depth studies are needed.

There are several limitations to our study. First, it was a convenience study precipitated by the use of probiotics for loose and non-infectious stools. As such, it is limited by small sample size but benefits from pair-wise study design. We observed significant differences in clinically relevant measures during synbiotic supplementation, suggesting our small sample size was nevertheless adequately powered. Similarly, following high fat diet challenge, we observed significant differences in chao 1 but not Shannon diversity measures. Given observed significance in some but not all measures, our findings most likely reflect the underlying biology and support the notion that synbiotic supplementation is not associated with persistent changes in the gut ecology. Second, we could not detect the probiotic strains in post-supplementation stool samples. However, this inability to detect the bacterial strains in the synbiotic per se are both consistent with and in contrast to previous human studies [29, 31, 34, 35, 41], and may be due to several reasons, such as synbiotic dose, probiotic species, and model system. For example, prior studies in humans have utilized *Bifidobacterium* [29, 31, 34, 35, 41], which differs from the probiotics used in this study. Thus, the inability to detect strains or species may be due to the absence of *Bifidobacteria* in the synbiotic supplement. However, we were able to detect alterations in *Lactobacillus* at the genus level in supplemented juveniles (Additional file 1: Figures S4 and S5). This lack of detection at the species level is consistent with other studies, and may be due to the complexity of the sample, the probiotic dosage, and/or model system. A prior study in canines where bacterial DNA from a probiotic was detected by quantitative real-time PCR (qPCR) provided daily supplementation at > 10^8^ CFU [86]. In contrast and consistent with our findings, a human study employing culturable strains similarly could only detect later stool output by qPCR at the genus level [87]. More recently, a study in preterm infants was able to detect probiotic strain DNA by qPCR but only in neonatal stool [88], which is non-complex and harbors similar strain microbes found within the NICU [89]. Therefore, the ability to detect probiotic bacterial DNA in the stool of preterm infants may be due a lower complexity of the sample or shared built environments.

Despite these limitations, our study has several strengths. First, it is a “real world” probiotic evaluation, whereby initial use followed common indications in both veterinary and human populations (namely, loose stools). Second, despite our inability to detect our probiotics strains (but demonstrated ability to detect same-strain genus increased abundance), we have observed significant improvement in lipid profiles concomitant with microbiome rearrangement and prokaryotic pathway enrichment unique to our synbiotic supplemented animals and only during supplementation. Thus, our data suggest that the probiotic mechanisms are likely through temporal ecological modulation rather than direct effects. Third, we leveraged an exceedingly well characterized primate model and were thus able to control for many potential confounders.

## Conclusions

Collectively, our findings in our primate model of maternal HFD feeding indicate that synbiotic supplementation is temporally associated with minimal and a few significant alterations in the gut microbiome of juvenile offspring. Overall, the majority of microbiome alterations in both beta and alpha diversity were not significant. However, synbiotic supplementation was affiliated with significant changes in the enrichment of specific carbohydrate pathways that may be tied to microbial production of SCFAs and host lipid profiles associated with both a maternal HFD and later in life HFD challenge [90]. Nevertheless, a protective effect against further gut dysbiosis associated with a HFD challenge at 2 years of age was not observed. Altogether, our data suggest that while synbiotic supplementation is associated with a few significant modulations of the microbiome and functional benefits to host physiology, this synbiotic-altered gut ecology does not persist and is refractory to later HFD feeding. These findings further support our prior observations that HFD exposure, be it solely maternal or as a later in life challenge, remains a significant driver of alterations of the gut microbiome. This lasting footprint of the maternal diet persists, and cannot be beneficially modified in the two years postnatally by neither synbiotic supplementation nor control diet feeding from the time of weaning onward.

## Additional files

Additional file 1: **Figure S1.** Loose stools and synbiotic supplementation was not associated with significant weight loss. Juveniles were supplemented with synbiotics after experiencing loose stools (*n* = 7). To determine if weight loss following symptoms and synbiotic supplementation occurred, we compared the weight of the synbiotic supplementation cohort with an untreated, healthy, age-matched cohort (*n* = 4) that was also matched for dietary exposures. Measurement of body weight was performed using the Ohaus ES series scale (Parsippany, NJ). There was no significant difference in the mean weight of synbiotic supplemented juveniles in comparison to untreated juveniles from prior, during, and post-supplementation. Error bars represent standard deviation. Test for significance was performed using Sidak’s multiple-comparisons test. (PDF 132 kb)

Additional file 2: **Table S1.** Sample/count summary. Provided are sequencing read counts (minimum, median, max, and mean) alongside sex, age and number of samples for each animal. SD = standard deviation. (PDF 24 kb)

Additional file 3: **Table S2.** Primers used in probiotic species-specific PCR. Table of primers used for PCR identification of probiotic species and/or genus. (PDF 21 kb)

Additional file 4: **Table S3.** Unweighted and weighted UniFrac PERMANOVA *p*-values of samples from untreated (15mo), pre-, mid-, and post-supplementation juveniles (1, 2, and 4 months after the end of supplementation). (PDF 12 kb)

Additional file 5: **Figure S2.** Bacterial taxa altered in association with synbiotic supplementation. LEfSe plot of bacterial taxa with differential abundance. Areas to the right and shaded orange indicate bacterial taxa with a higher abundance in animals from the pre-supplementation group, whereas areas to the left and shaded red indicate bacterial taxa with a higher abundance in animals from the untreated (15 months) group. (PDF 342 kb)

Additional file 6: **Figure S3.** Taxonomic shifts associated with synbiotic supplementation. **A**. LEfSe plot of bacterial genera with an increased relative abundance during pre-supplementation. LEfSe did not detect an enrichment of bacterial taxa during mid-supplementation. **B**. Examination of the relative abundance of probiotic strains from pre-supplementation to 4-months post-supplementation revealed no significant changes. Statistical significance was tested using Dunn’s multiple comparisons test. (PDF 406 kb)

Additional file 7: **Table S4.** BLAST results for OTU representative sequences. (XLSX 12 kb)

Additional file 8: **Figure S4.** Majority of species-level bacterial DNA from probiotics is undetectable in the gut of supplemented juveniles. DNA was isolated from anal swabs and stool samples collected at pre-, mid-, post-supplementation. PCR was performed to detect probiotic bacterial DNA at the species level (*Enterococcus faecium, Lactobacillus plantarum*, *L. casei*, and *L. acidophilus*). *L. acidophilus* was the only probiotic bacterial species that was detected at any time point assayed, and only detected in 2 animals (animal 31,267 and faint band in 31,093). A 16S universal PCR was performed to ensure the presence of bacteria within the samples, and species specific positive controls were run alongside blank negative controls (far right panel). *n* = 7 subjects per time point. (PDF 19 kb)

Additional file 9: **Figure S5.** *Lactobacillus* genera are quantitatively increased in the gut of synbiotic-supplemented juveniles. DNA was isolated from anal swabs and stool samples collected at pre-, mid-, post-supplementation. PCR was performed to detect *Lactobacillus* at the genus level. (A) While bacterial DNA from probiotics was undetectable at the species level in the gut of supplemented juveniles, we were able to detect *Lactobacillus* in the gut at the genus level in both pre-, mid, and post supplemented juveniles. (B) Increases in *Lactobacillus* at the genus level was detectable by quantitative real-time PCR (qPCR). (C) The majority of supplemented juveniles (5/7) had a detectable increase in *Lactobacillus* at the genus level by qPCR, which resolved post-supplementation. *n* = 7 subjects per time point. (PDF 5089 kb)

Additional file 10: **Figure S6.** Relative abundance of LEfSe-identified enriched KEGG pathways in the pre-supplemented and mid synbiotic supplemented groups. STAMP generated two groups extended error bar plot. Plots on the left indicate relative abundance of enriched KEGG pathways. Plot on the right indicates the difference in the mean proportion. Statistical significance was tested using White’s non-parametric t-test. (PDF 260 kb)

Additional file 11: **Table S5.** Unweighted and weighted UniFrac PERMANOVA p-values of samples from juveniles before and during the HFD challenge (un15mo, un26mo, P4M, HFD). (PDF 12 kb)

Additional file 12: **Figure S7.** Alterations in bacterial taxa and metabolic pathways are associated with HFD challenge. **A**. LEfSe generated cladogram of taxonomic differences of abundance. Areas shaded blue indicate bacterial taxa with a higher abundance in prior synbiotic supplemented animals at 4-months post- supplementation, whereas areas shaded purple indicate bacterial taxa with a higher abundance in prior synbiotic supplemented animals during HFD challenge. **B**. LEfSe plot of bacterial metabolic pathways with differential abundance. Areas to the right and shaded blue indicate bacterial metabolic pathways enriched in synbiotic-supplemented animals at 4-months post-synbiotic, whereas areas to the left and shaded purple indicate bacterial metabolic pathways enriched in prior synbiotic supplemented animals during HFD challenge. **C**. LEfSe generated cladogram of taxonomic differences of abundance. Areas shaded blue indicate bacterial taxa with a higher abundance in prior synbiotic supplemented animals at 4-months post-treatment, whereas areas shaded green indicate bacterial taxa with a higher abundance in untreated animals during HFD challenge. **D**. LEfSe plot of bacterial metabolic pathways with differential abundance. Areas to the right and shaded blue indicate inferred bacterial metabolic pathways enriched in synbiotic supplemented animals at 4-months post-treatment, whereas areas to the left and shaded green indicate bacterial metabolic pathways enriched in untreated animals during HFD challenge. (PDF 3901 kb)

Additional file 13: **Figure S8.** Relative abundance of LEfSe-identified enriched KEGG pathways in the synbiotic supplemented and untreated groups during HFD challenge. STAMP generated two groups extended error bar plot. Plots on the left indicate relative abundance of enriched KEGG pathways. Plot on the right indicates the difference in the mean proportion. Statistical significance was tested using White’s non-parametric t-test. (PDF 461 kb)

## Abbreviations

HFD: High-fat diet
NAFLD: Non-alcoholic fatty liver disease
NHP: Non-human primate
OTU: Operational Taxonomic Unit
SCFA: Short-chain fatty acid
