# Supplementary figures and images for: Modulations in the offspring gut microbiome are refractory to postnatal synbiotic supplementation among juvenile primates
Source: BMC Microbiol. 2018 Apr 5;18:28. doi: 10.1186/s12866-018-1169-9 (PMC5887201; doi:10.1186/s12866-018-1169-9)

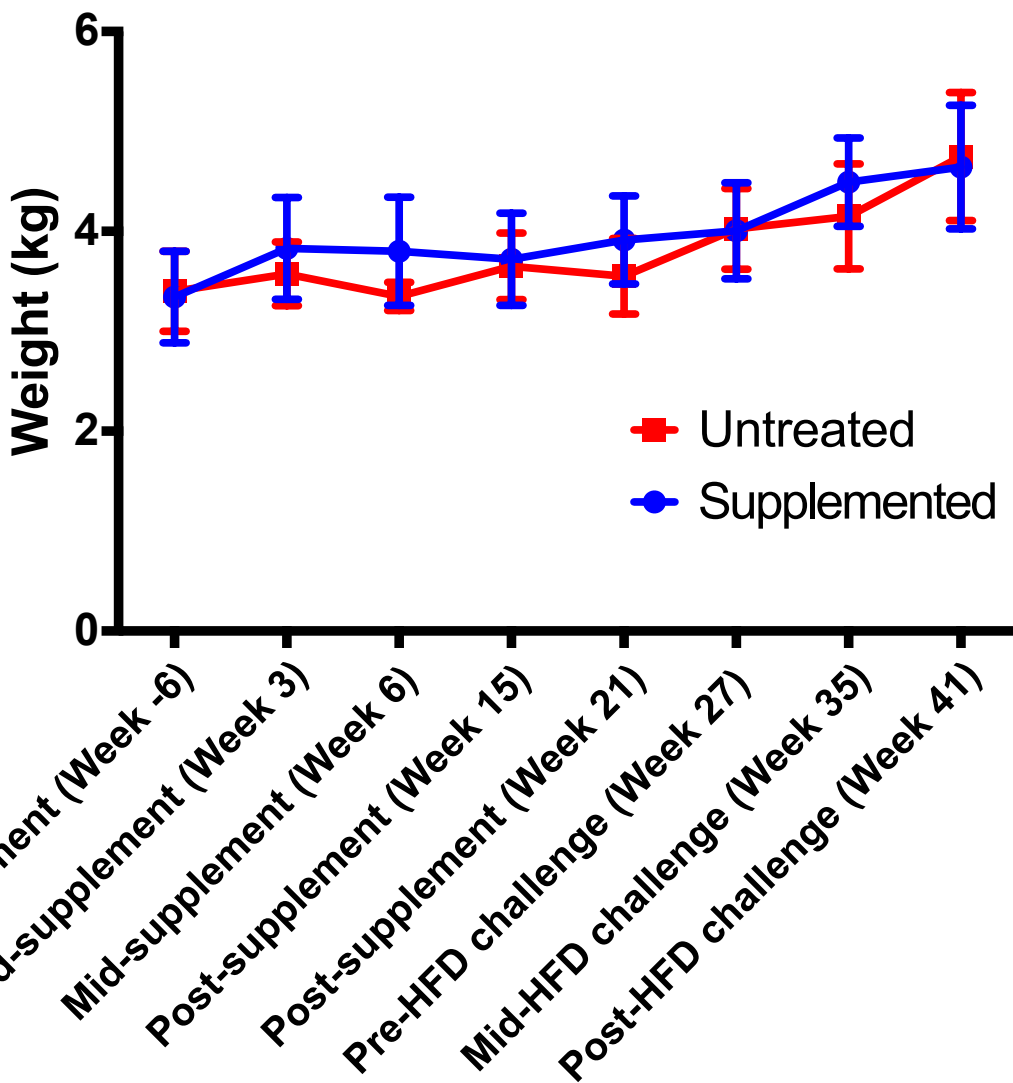

Supplement: Supplementary file 1 — Figure S1. Loose stools and synbiotic supplementation was not associated with significant weight loss. Juveniles were supplemented with synbiotics after experiencing loose stools (n = 7). To determine if weight loss following symptoms and synbiotic supplementation occurred, we compared the weight of the synbiotic supplementation cohort with an untreated, healthy, age-matched cohort (n = 4) that was also matched for dietary exposures. Measurement of body weight was performed using the Ohaus ES series scale (Parsippany, NJ). There was no significant difference in the mean weight of synbiotic supplemented juveniles in comparison to untreated juveniles from prior, during, and post-supplementation. Error bars represent standard deviation. Test for significance was performed using Sidak’s multiple-comparisons test. (PDF 132 kb) [file 12866_2018_1169_MOESM1_ESM.pdf]

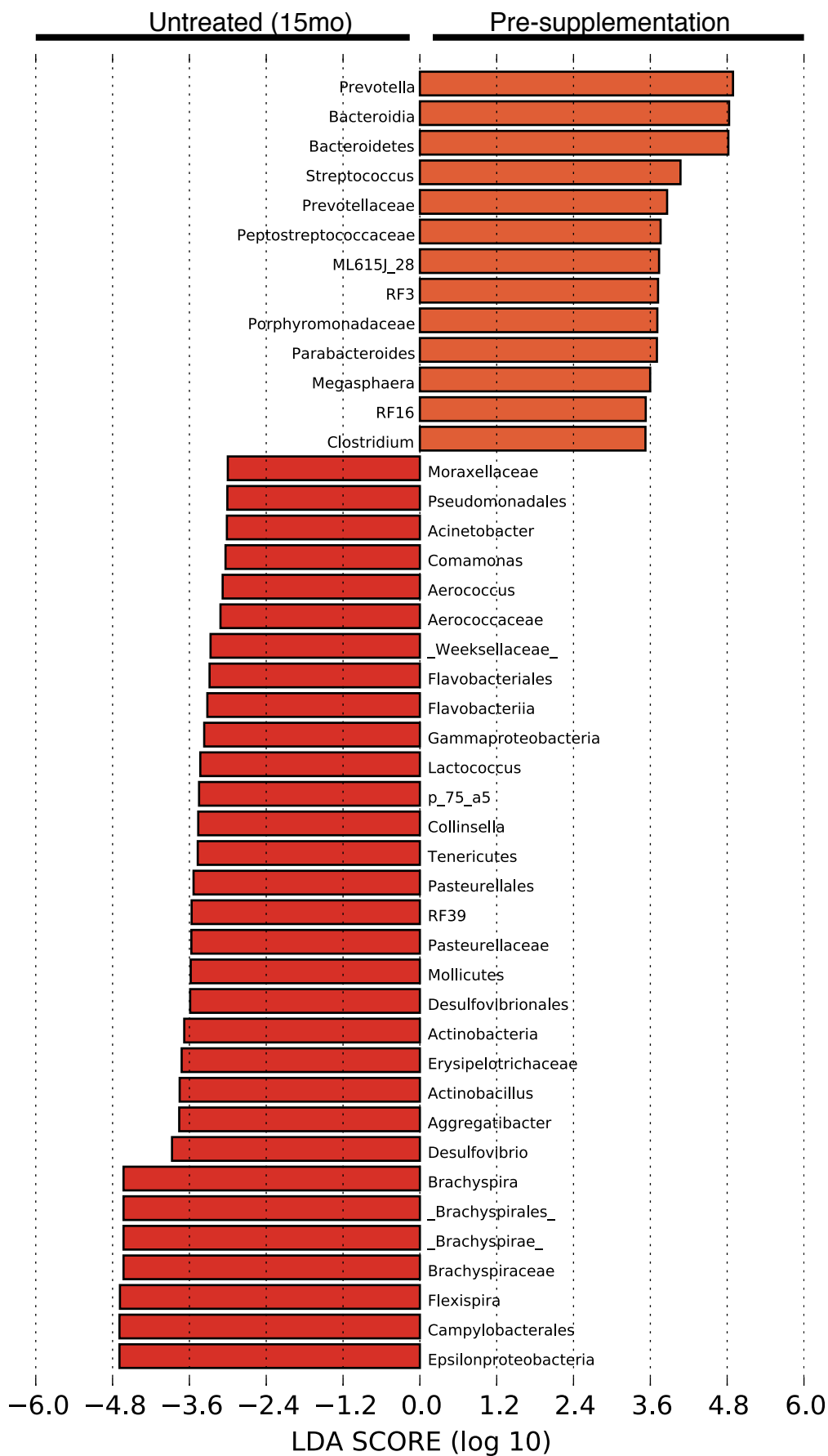

Supplement: Supplementary file 5 — Figure S2. Bacterial taxa altered in association with synbiotic supplementation. LEfSe plot of bacterial taxa with differential abundance. Areas to the right and shaded orange indicate bacterial taxa with a higher abundance in animals from the pre-supplementation group, whereas areas to the left and shaded red indicate bacterial taxa with a higher abundance in animals from the untreated (15 months) group. (PDF 342 kb) [file 12866_2018_1169_MOESM5_ESM.pdf]

A

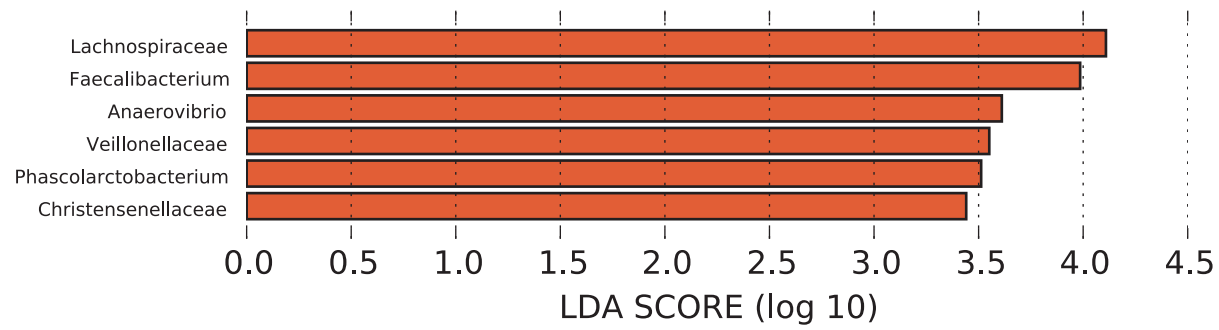

B

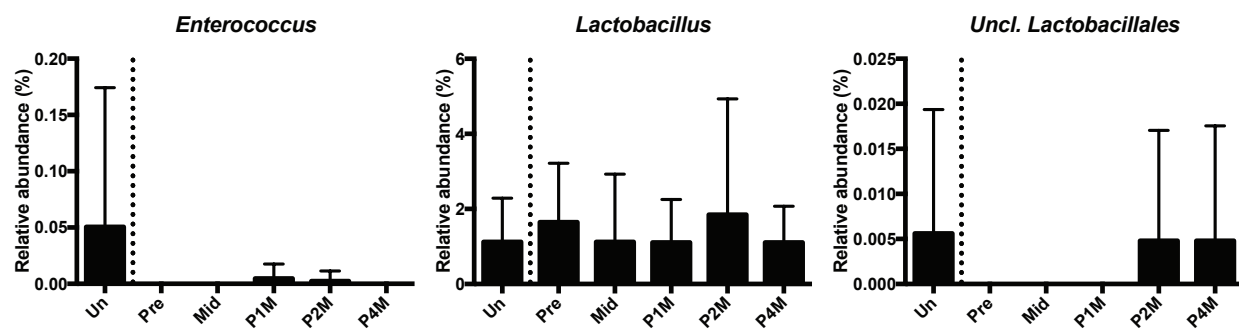

Supplement: Supplementary file 6 — Figure S3. Taxonomic shifts associated with synbiotic supplementation. A. LEfSe plot of bacterial genera with an increased relative abundance during pre-supplementation. LEfSe did not detect an enrichment of bacterial taxa during mid-supplementation. B. Examination of the relative abundance of probiotic strains from pre-supplementation to 4-months post-supplementation revealed no significant changes. Statistical significance was tested using Dunn’s multiple comparisons test. (PDF 406 kb) [file 12866_2018_1169_MOESM6_ESM.pdf]

**A**

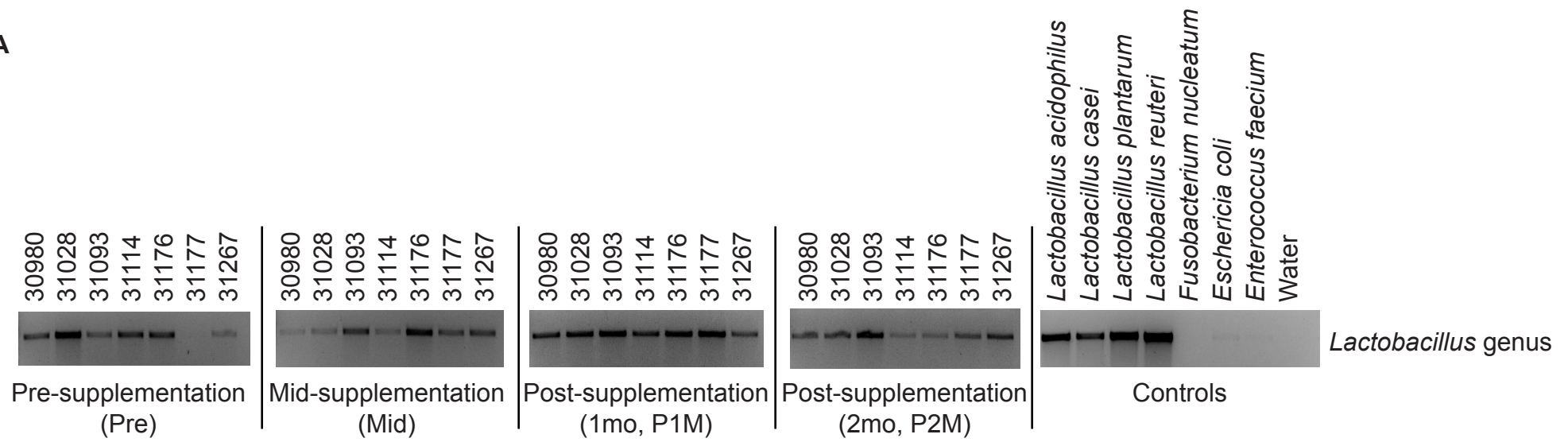

**B**

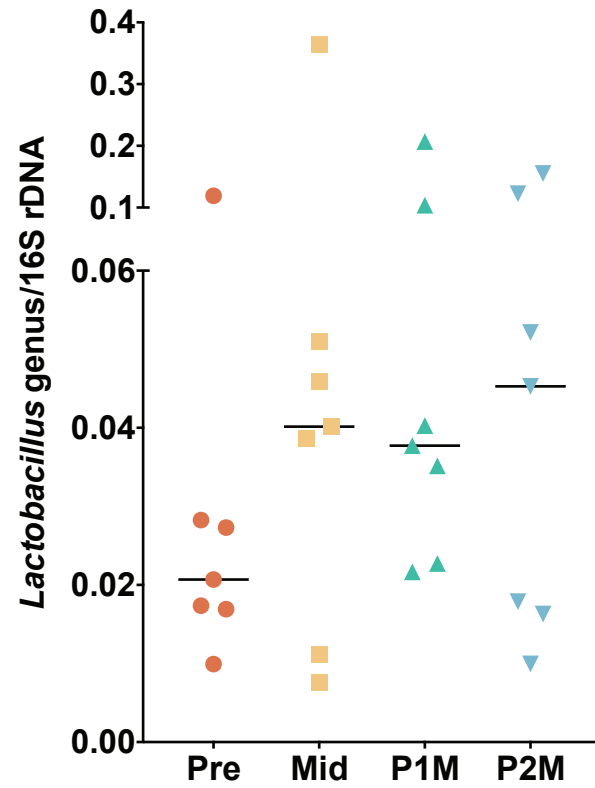

**C**

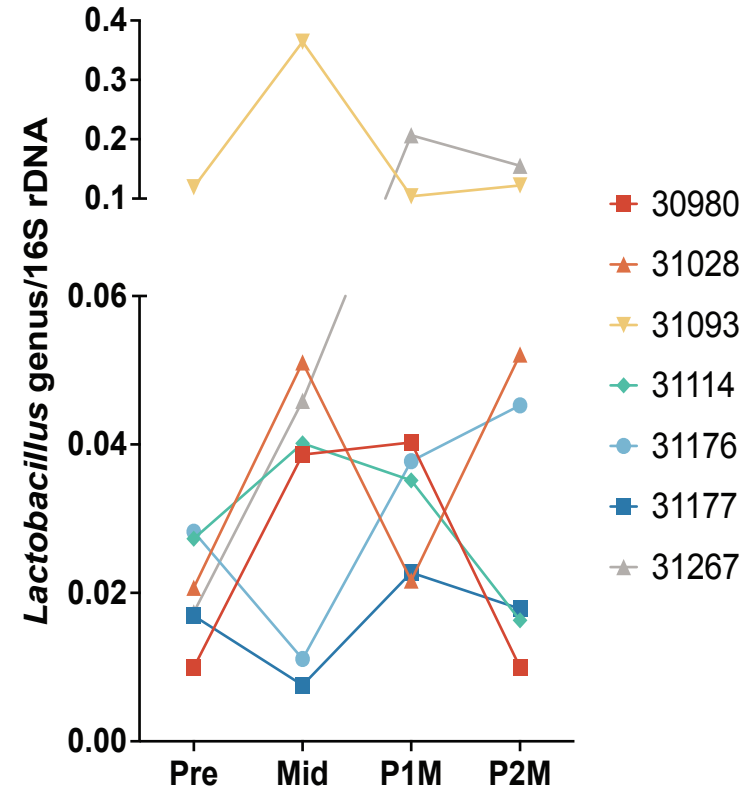

Supplement: Supplementary file 9 — Figure S5. Lactobacillus genera are quantitatively increased in the gut of synbiotic-supplemented juveniles. DNA was isolated from anal swabs and stool samples collected at pre-, mid-, post-supplementation. PCR was performed to detect Lactobacillus at the genus level. (A) While bacterial DNA from probiotics was undetectable at the species level in the gut of supplemented juveniles, we were able to detect Lactobacillus in the gut at the genus level in both pre-, mid, and post supplemented juveniles. (B) Increases in Lactobacillus at the genus level was detectable by quantitative real-time PCR (qPCR). (C) The majority of supplemented juveniles (5/7) had a detectable increase in Lactobacillus at the genus level by qPCR, which resolved post-supplementation. n = 7 subjects per time point. (PDF 5089 kb) [file 12866_2018_1169_MOESM9_ESM.pdf]

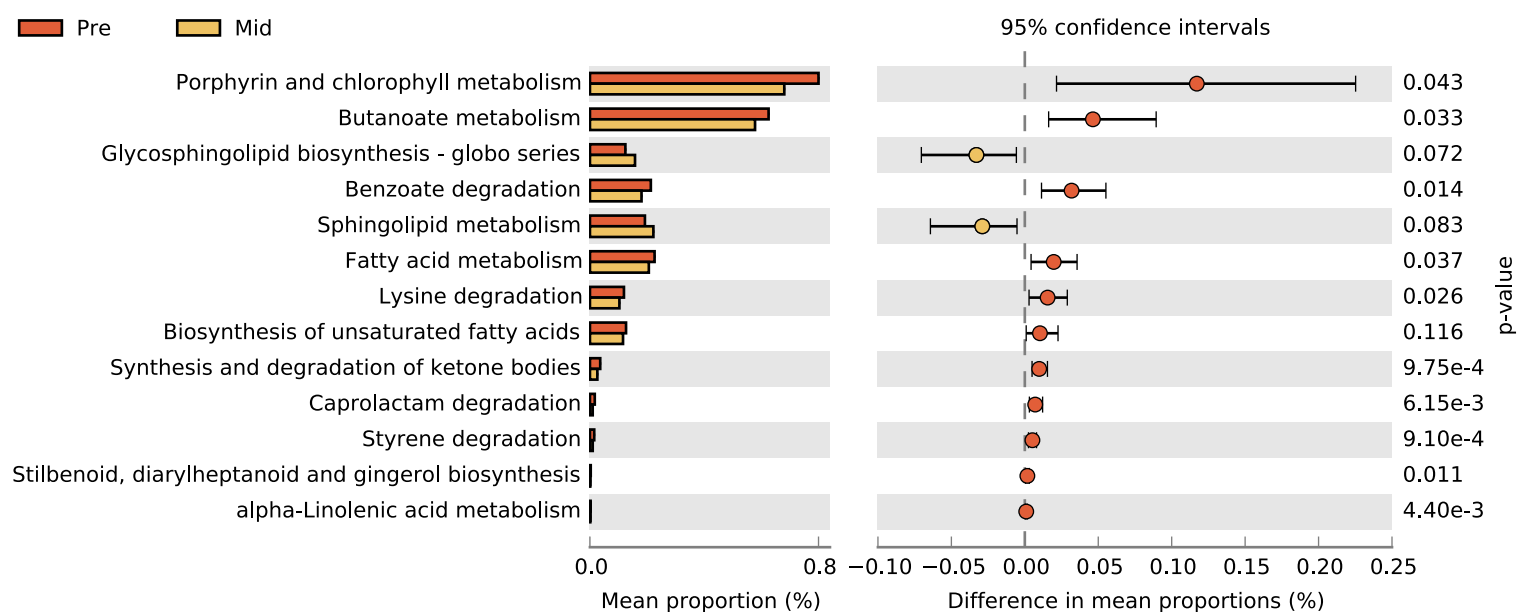

Supplement: Supplementary file 10 — Figure S6. Relative abundance of LEfSe-identified enriched KEGG pathways in the pre-supplemented and mid synbiotic supplemented groups. STAMP generated two groups extended error bar plot. Plots on the left indicate relative abundance of enriched KEGG pathways. Plot on the right indicates the difference in the mean proportion. Statistical significance was tested using White’s non-parametric t-test. (PDF 260 kb) [file 12866_2018_1169_MOESM10_ESM.pdf]

A

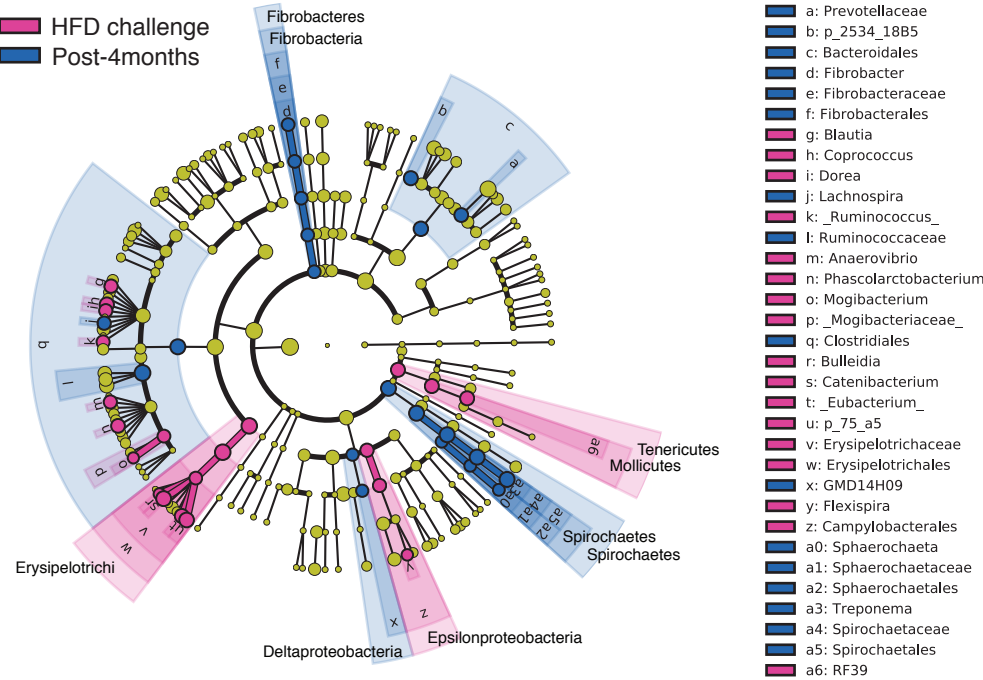

B

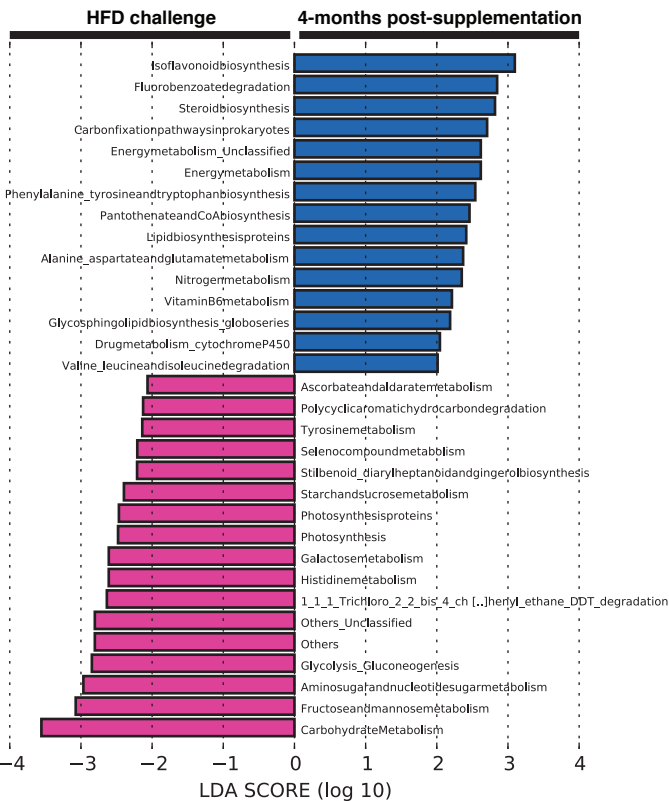

C

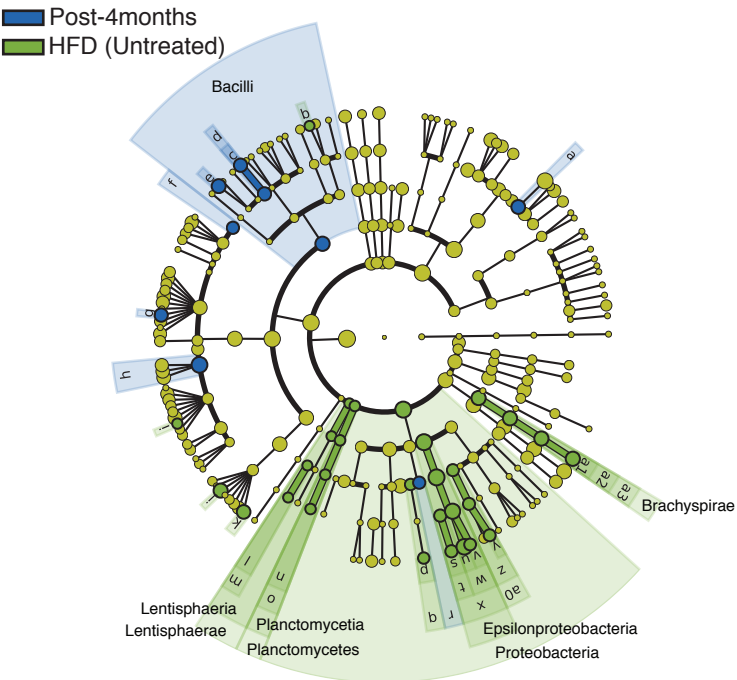

D

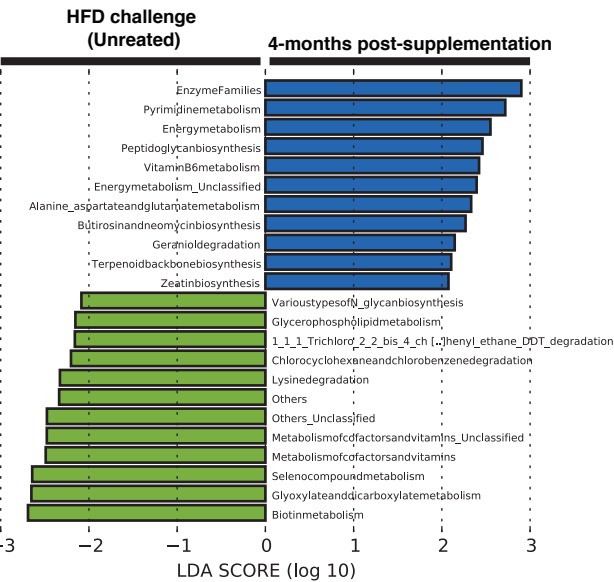

Supplement: Supplementary file 12 — Figure S7. Alterations in bacterial taxa and metabolic pathways are associated with HFD challenge. A. LEfSe generated cladogram of taxonomic differences of abundance. Areas shaded blue indicate bacterial taxa with a higher abundance in prior synbiotic supplemented animals at 4-months post- supplementation, whereas areas shaded purple indicate bacterial taxa with a higher abundance in prior synbiotic supplemented animals during HFD challenge. B. LEfSe plot of bacterial metabolic pathways with differential abundance. Areas to the right and shaded blue indicate bacterial metabolic pathways enriched in synbiotic-supplemented animals at 4-months post-synbiotic, whereas areas to the left and shaded purple indicate bacterial metabolic pathways enriched in prior synbiotic supplemented animals during HFD challenge. C. LEfSe generated cladogram of taxonomic differences of abundance. Areas shaded blue indicate bacterial taxa with a higher abundance in prior synbiotic supplemented animals at 4-months post-treatment, whereas areas shaded green indicate bacterial taxa with a higher abundance in untreated animals during HFD challenge. D. LEfSe plot of bacterial metabolic pathways with differential abundance. Areas to the right and shaded blue indicate inferred bacterial metabolic pathways enriched in synbiotic supplemented animals at 4-months post-treatment, whereas areas to the left and shaded green indicate bacterial metabolic pathways enriched in untreated animals during HFD challenge. (PDF 3901 kb) [file 12866_2018_1169_MOESM12_ESM.pdf]

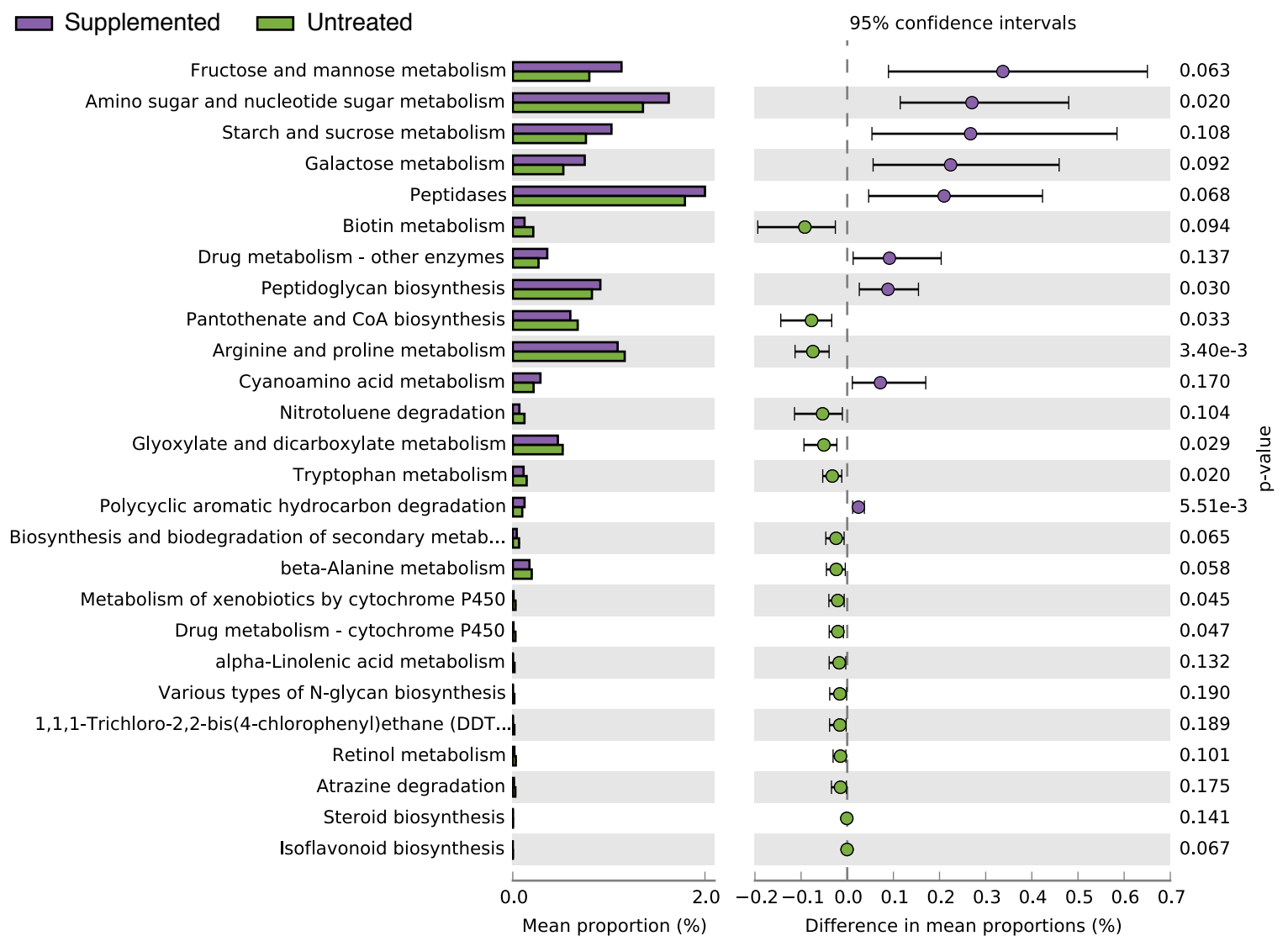

Supplement: Supplementary file 13 — Figure S8. Relative abundance of LEfSe-identified enriched KEGG pathways in the synbiotic supplemented and untreated groups during HFD challenge. STAMP generated two groups extended error bar plot. Plots on the left indicate relative abundance of enriched KEGG pathways. Plot on the right indicates the difference in the mean proportion. Statistical significance was tested using White’s non-parametric t-test. (PDF 461 kb) [file 12866_2018_1169_MOESM13_ESM.pdf]
