# Supplementary material for: Modulations in the offspring gut microbiome are refractory to postnatal synbiotic supplementation among juvenile primates
Source: BMC Microbiol. 2018 Apr 5;18:28. doi: 10.1186/s12866-018-1169-9 (PMC5887201; doi:10.1186/s12866-018-1169-9)
Supplement: Supplementary file 2 — Table S1. Sample/count summary. Provided are sequencing read counts (minimum, median, max, and mean) alongside sex, age and number of samples for each animal. SD = standard deviation. (PDF 24 kb) [file 12866_2018_1169_MOESM2_ESM.pdf]

		Untreated, 15 months (anus & stool)	Untreated & HFD Challenge, 26 months (anus)	Pre-supplementation (anus)	Mid-supplementation (stool)	1-month post-supplementation (stool)	2-month post-supplementation (anus & stool)	4-months post-supplementation (stool)	HFD Challenge (stool)
**Juveniles (n)**		3	4	7	7	7	7	7	7
**Male (n)**		2	-	5	5	5	5	5	5
**Female (n)**		1	4	2	2	2	2	2	2
**Total samples (n)**		6	4	7	7	7	14	7	7
**Samples male (n)**		4	-	5	5	5	10	5	5
**Samples female (n)**		2	4	2	2	2	4	2	2
**Age at sampling (months)**		15	25-27	17-20	19-22	21-24	22-25	24-27	26-28
**Read counts** (Pre-filtered)	**Minimum**	15039	7717	3634	3932	3621	3835	3951	5242
	**Median**	22994	8845	5147	4986	5126	4481	4804	6474
	**Max**	30355	9846	6569	5762	5909	6780	7210	7777
	**Mean**	22508	8813	4989	4907	4839	4793	4941	6540
	**SD**	5610	902	924	673	900	857	1074	945
**Read counts** (post-closed referecne OTU picking)	**Minimum**	8700	4314	3269	3371	3135	2969	3207	4697
	**Median**	12335	6797	4366	4279	4324	3889	4017	5211
	**Max**	17281	7498	5841	5376	4998	5932	6404	6581
	**Mean**	12630	6351.5	4367	4176	4176	4090	4299	5557
	**SD**	2899	1453	842	791	783	861	1024	727
