# Supplementary material for: Modulations in the offspring gut microbiome are refractory to postnatal synbiotic supplementation among juvenile primates
Source: BMC Microbiol. 2018 Apr 5;18:28. doi: 10.1186/s12866-018-1169-9 (PMC5887201; doi:10.1186/s12866-018-1169-9)
Supplement: Supplementary file 3 — Table S2. Primers used in probiotic species-specific PCR. Table of primers used for PCR identification of probiotic species and/or genus. (PDF 21 kb) [file 12866_2018_1169_MOESM3_ESM.pdf]

Primer	Specificity	Sequence	Annealing Temperature	Reference
Lplant_For	*Lactobacillus plantarum*	TTA ATT TTC CGC GCA ACA AGT	57.5^{o}C	Perez-Diaz *et al* 2013
Lplant_Rev	*Lactobacillus plantarum*	CTA AAA GCG AAG TAT GGC GTC AAA	57.5^{o}C	Perez-Diaz *et al* 2013
LacidF1	*Lactobacillus acidophilus*	GAC TGA ATA CGA TTA CCC TGG TGAT	50.3 ^{o}C	Current study
LacidR1	*Lactobacillus acidophilus*	CTT ATG AGT TTG GAT TGA GCC G	50.3 ^{o}C	Current study
EM1A	*Enterococcus faecium*	TTG AGG CAG ACC AGA TTG ACG	57°C	Cheng *et al* 1997
EM1B	*Enterococcus faecium*	TAT GAC AGC GAC TCC GAT TCC	57°C	Cheng *et al* 1997
LcaseiF10	*Lactobacillus casei*	ACT GAT CGT GCC AAG GGT G	54°C	Current study
LcaseiR10	*Lactobacillus casei*	TAG GCT TCA ACA CCT GGC TG	54°C	Current study
LactoF1	*Lactobaccillus* genus	GCA TGG GTA GCG AAC AGG	54°C	Current study
LactoR1	*Lactobaccillus* genus	CAC CTT CCT CCG GTT TGT CA	54°C	Current study
F_alllact_IS	*Lactobacillus*	TGG ATG CCT TGG CAC TAG GA	53°C	Haarman and Knol 2006
R_alllact_IS	*Lactobacillus*	AAA TCT CCG GAT CAA AGC TTA CTT AT	53°C	Haarman and Knol 2006
16S-Universal-334-F	16S Universal	TCC TAC GGG AGG CAG CAC T	60°C	Nadkarni *et al* 2002, Mohammadi *et al* 2003, Ma *et al* 2014
16S-Universal-800-R	16S Universal	GGA CTA CCA GGG TAT CTA ATC CTG TT	60°C	Nadkarni *et al* 2002, Mohammadi *et al* 2003, Ma *et al* 2014
