# Supplementary material for: Modulations in the offspring gut microbiome are refractory to postnatal synbiotic supplementation among juvenile primates
Source: BMC Microbiol. 2018 Apr 5;18:28. doi: 10.1186/s12866-018-1169-9 (PMC5887201; doi:10.1186/s12866-018-1169-9)
Supplement: Supplementary file 4 — Table S3. Unweighted and weighted UniFrac PERMANOVA p-values of samples from untreated (15mo), pre-, mid-, and post-supplementation juveniles (1, 2, and 4 months after the end of supplementation). (PDF 12 kb) [file 12866_2018_1169_MOESM4_ESM.pdf]

	Unweighted UniFrac	Weighted UniFrac
**Sex**	0.093	0.328
**Age at sample (months)**	0.001	0.094
**Sample group**	0.001	0.046
**Received synbiotics** (untreated vs. synbiotic supplemented)	0.001	0.005
