# Supplementary material for: Modulations in the offspring gut microbiome are refractory to postnatal synbiotic supplementation among juvenile primates
Source: BMC Microbiol. 2018 Apr 5;18:28. doi: 10.1186/s12866-018-1169-9 (PMC5887201; doi:10.1186/s12866-018-1169-9)
Supplement: Supplementary file 8 — Figure S4. Majority of species-level bacterial DNA from probiotics is undetectable in the gut of supplemented juveniles. DNA was isolated from anal swabs and stool samples collected at pre-, mid-, post-supplementation. PCR was performed to detect probiotic bacterial DNA at the species level (Enterococcus faecium, Lactobacillus plantarum, L. casei, and L. acidophilus). L. acidophilus was the only probiotic bacterial species that was detected at any time point assayed, and only detected in 2 animals (animal 31,267 and faint band in 31,093). A 16S universal PCR was performed to ensure the presence of bacteria within the samples, and species specific positive controls were run alongside blank negative controls (far right panel). n = 7 subjects per time point. (PDF 19 kb) [file 12866_2018_1169_MOESM8_ESM.pdf]

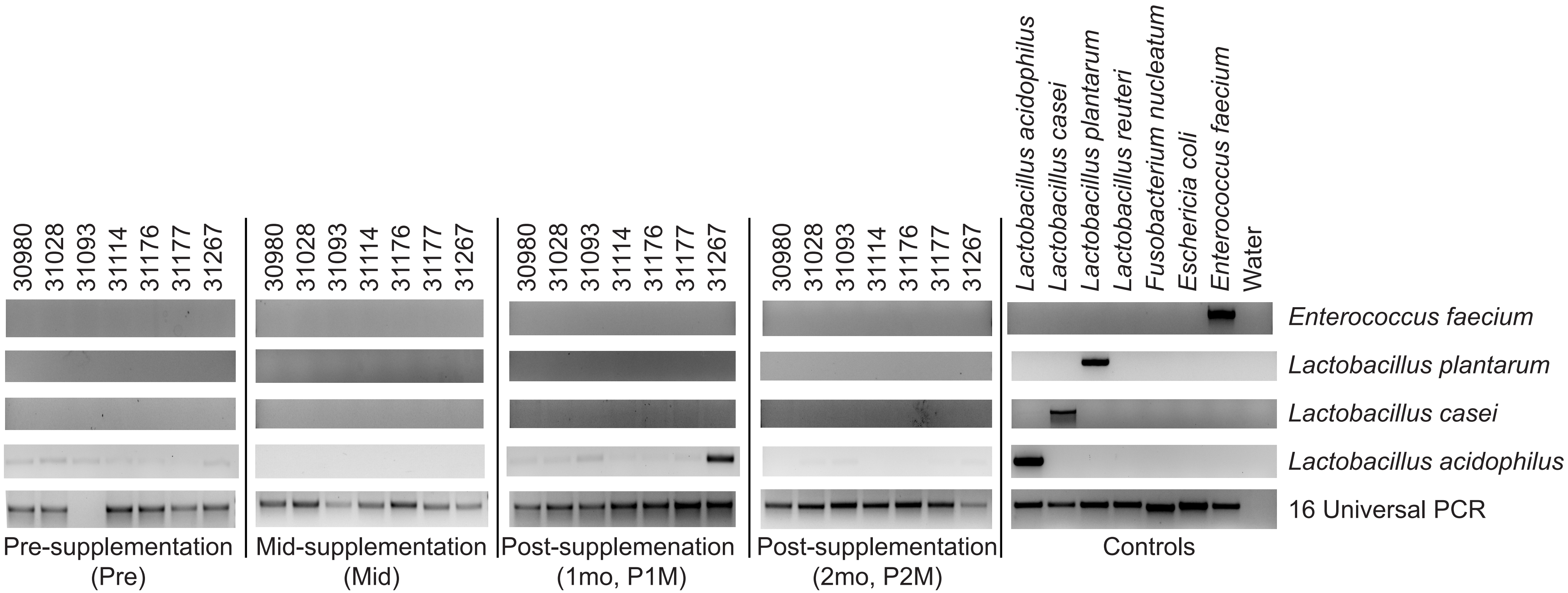
30980
31028
31093
31114
31176
31177
31267
Pre-supplementation (Pre)
Mid-supplementation (Mid)
Post-supplemenation (1mo, P1M)
Post-supplementation (2mo, P2M)
Lactobacillus acidophilus
Lactobacillus casei
Lactobacillus plantarum
Lactobacillus reuteri
Fusobacterium nucleatum
Eschericia coli
Enterococcus faecium
Water
Controls
Enterococcus faecium
Lactobacillus plantarum
Lactobacillus casei
Lactobacillus acidophilus
16 Universal PCR
