# Supplementary material for: Modulations in the offspring gut microbiome are refractory to postnatal synbiotic supplementation among juvenile primates
Source: BMC Microbiol. 2018 Apr 5;18:28. doi: 10.1186/s12866-018-1169-9 (PMC5887201; doi:10.1186/s12866-018-1169-9)
Supplement: Supplementary file 11 — Table S5. Unweighted and weighted UniFrac PERMANOVA p-values of samples from juveniles before and during the HFD challenge (un15mo, un26mo, P4M, HFD). (PDF 12 kb) [file 12866_2018_1169_MOESM11_ESM.pdf]

	Unweighted UniFrac	Weighted UniFrac
**Sex**	0.559	0.663
**Age at sample (months)**	0.056	0.359
**Sample group**	0.001	0.024
**Prior synbiotic supplementation**	0.001	0.022
**HFD challenge**	0.001	0.066
